# Thoracoscopic Right S^4^ Segmentectomy for Pulmonary Arteriovenous Fistula Involving an Aberrant Bronchus: A Case Report

**DOI:** 10.70352/scrj.cr.26-0467

**Published:** 2026-09-11

**Authors:** Hirohisa Kato, Masami Abiko

**Affiliations:** Department of Thoracic Surgery, Okitama Public General Hospital, Higashiokitama-gun, Yamagata, Japan

**Keywords:** pulmonary arteriovenous fistula, indocyanine green, near-infrared fluorescent, thoracoscopic segmentectomy, aberrant bronchus, simulation

## Abstract

**INTRODUCTION:**

Although pulmonary arteriovenous fistulas (PAVFs) have often been treated with embolization, surgical approaches including segmentectomy might also play a curative role. Furthermore, a near-infrared fluorescent (NIF) imaging has been widely used as an assisting tool during surgery and has been useful for identifying the intersegmental plane during pulmonary segmentectomy. We describe a thoracoscopic right S^4^ segmentectomy for a PAVF case involving a rare aberrant bronchus in the middle lobe bronchus using NIF imaging to identify the intersegmental plane and target the segmental bronchus.

**CASE PRESENTATION:**

A 66-year-old woman presented with a PAVF consisting of dilated feeding and draining vessels (A^4^ and V^4^) in the right S^4^, the percutaneous oxygen saturation was 94%, and thoracoscopic right S^4^ segmentectomy was planned. Preoperatively, a bronchial CT simulation revealed a middle lobe bronchus involving a rare aberrant bronchus (B^7^a), and the target segmental bronchus (B^4^) was bronchoscopically marked with indocyanine green (ICG). Intraoperatively, the B^4^ was identified with NIF imaging and divided following division of the dilated vessels. The PAVF was completely removed with the dilated vessels, while the aberrant bronchus was preserved.

**CONCLUSIONS:**

Segmentectomy can contribute to a curative resection of PAVF, bronchial CT simulation helps to recognize the variant bronchus, and NIF imaging can identify not only the intersegmental plane but also the target segmental bronchus for division in PAVF cases with aberrant bronchus requiring thoracoscopic segmentectomy.

## Abbreviations

AVF
: arteriovenous fistula
ICG
: indocyanine green
NIF
: near-infrared fluorescent
PAVF
: pulmonary arteriovenous fistula

## INTRODUCTION

Although most PAVFs have recently been treated with embolizations using materials like coils, plug, and balloon, surgical treatment can also be performed.^1,2)^ The surgical approaches include wedge resection, segmentectomy, lobectomy, pneumonectomy, and fistulectomy; these procedures are selected based on characteristics including the isolated lesion or multiple lesions and lesion size.^3,4)^ Among these, segmentectomy has been increasingly performed in patients with lung cancer since 2 clinical trials revealed the non-inferiority of segmentectomy compared to lobectomy for lung cancer with less than 2 cm tumor diameter, and the advantages of anatomical resection and preserving pulmonary function, although it requires a precise anatomical understanding and some technical skills due to complexities.^5,6)^ A NIF imaging method using ICG has been widely used as a procedural assistance during surgery, and the usefulness has been broadly recognized to be acceptable for identifying the intersegmental planes during segmentectomy.^7)^ Furthermore, it is occasionally confusing to identify the target segmental bronchus during the procedure; we recently devised a novel method for identifying the target segmental bronchus via NIF imaging using ICG after approval by the Institutional Review Board.^8)^ Herein, we present a PAVF case that underwent thoracoscopic segmentectomy using the abovementioned method.

## CASE PRESENTATION

**Fig. 1:**
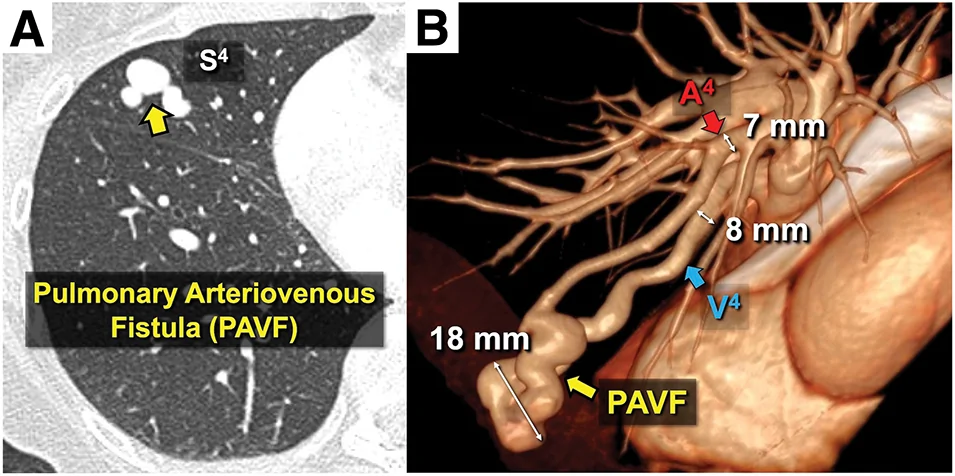
PAVF on preoperative CT and 3D reconstruction images. (**A**) The PAVF was shown on right S^4^. (**B**) PAVF was constructed with A^4^ and V^4^, which were dilated to 7 and 8 mm in diameter, respectively. PAVF, pulmonary arteriovenous fistula

**Fig. 2:**
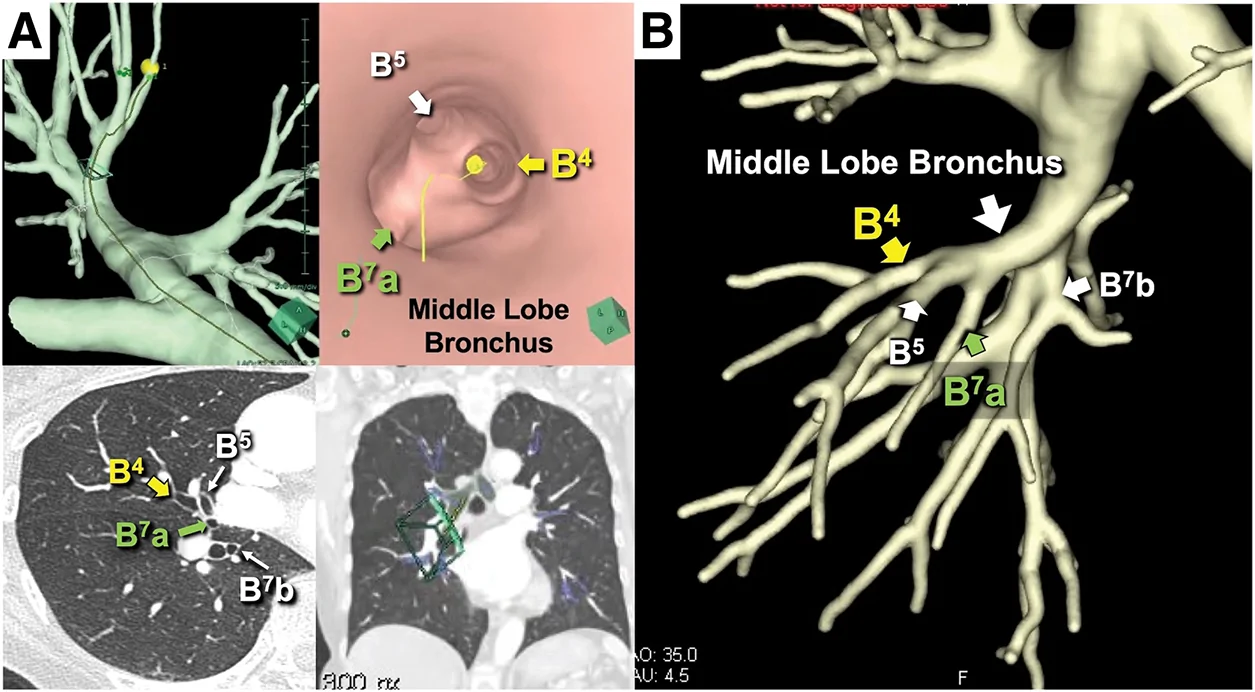
Preoperative bronchial CT simulation. (**A**) Aberrant bronchus (B^7^a) was recognized in the middle lobe bronchus. (**B**) B^7^a diverged from the middle lobe bronchus on preoperative 3D reconstruction image of the bronchial tree.

A 66-year-old woman presented with a PAVF in the right lateral segment (S^4^) of the middle lobe on CT (**Fig. 1A**). Percutaneous oxygen saturation was 94% at rest, and the patient felt slight exertional dyspnea. 3D CT imaging (Vue PACS; Philips Japan, Tokyo, Japan) demonstrated a PAVF 18 mm in size consisting of a feeding artery (A^4^) and a drainage vein (V^4^) dilated to 7 and 8 mm in diameter, respectively (**Fig. 1B**). Other AVF(arteriovenous fistulas) lesions were not identified outside the right middle lobe; the PAVF was diagnosed as a simple type. Thoracoscopic right S^4^ segmentectomy was planned, as the patient desired a curative surgery following a consultation with a radiologist. Preoperative bronchial CT simulation using the Synapse Vincent system (Fujifilm, Tokyo, Japan) revealed a rare aberrant bronchus (B^7^a) arising from the middle lobe bronchus and supplying the medial basal segment (**Fig. 2A** and **2B**).

Immediately before surgery, the target segmental bronchus (B^4^) was bronchoscopically marked by applying ICG (1.25 mg/mL)-soaked cotton that was placed in contact with the bronchial epithelium for several seconds. The ICG-soaked cotton was prepared by cutting a commercial-grade cotton (Osaki Medical, Nagoya, Japan) into 5-mm pieces and then soaking the cotton pieces in a reagent mixture of 2 mL (2.5 mg/mL) ICG and 2 mL Xylocaine 2% Jelly (Sandoz Pharma, Tokyo, Japan). Thereafter, bronchial marking was performed after tracheal intubation with a double-lumen tube under general anesthesia, with the patient in the supine position. The mucosa of the segmental bronchus (B^4^) was marked by swabbing the mucosa with the ICG-soaked cotton, which was wedged into the entire bronchial circumference using bronchoscopic forceps (FB-456D; Olympus Corporation, Tokyo, Japan) and a bronchoscope (Ambu aScope 4; Ambu, Tokyo, Japan). Afterward, the ICG-soaked cotton was removed (**Video 1**). The use of ICG for bronchial marking was approved by the Institutional Review Board of Okitama Public General Hospital (approval number 289, November 18, 2022). Informed consent was obtained from the patient under a clinical research protocol.

**Fig. 3:**
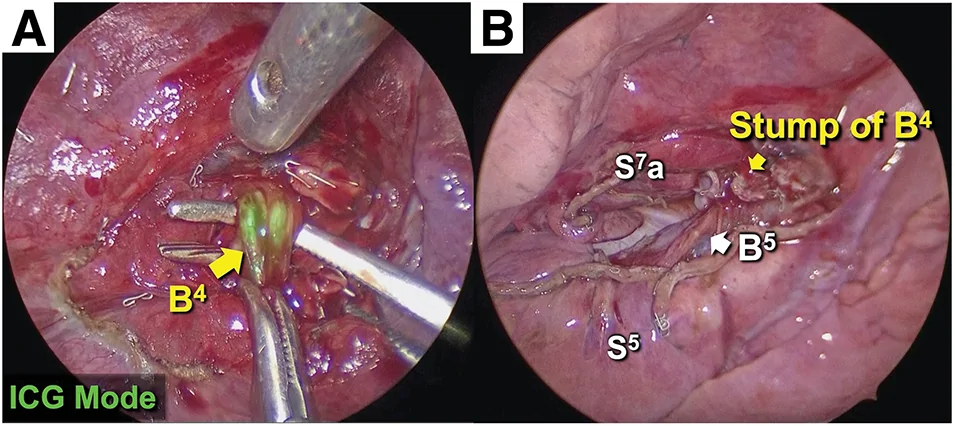
Intraoperative findings. (**A**) The target segmental bronchus (B^4^) was clearly identified with NIF image. (**B**) Adjacent bronchus (B^5^) and segments (S^5^ and S^7^a) were preserved. NIF, near-infrared fluorescent

During surgery, the corresponding vessels, V^4^ and A^4^, were carefully dissected and divided sequentially through 4-access ports; the B^4^ was clearly identified under the thoracoscopic ICG mode and divided (**Fig. 3A** and **3B**). After the intersegmental division between S^4^ and S^7^a, the intersegmental plane was identified using NIF imaging after intravenously injecting ICG (10 mg), and the inflation–deflation line was created by inflation of the adjacent segment (S^5^). The intersegmental parenchyma between S^4^ and S^5^ was divided, and the PAVF was removed (**Video 1**). Surgical time was 143 minutes, and blood loss was minimal. The drainage period and hospital stay were 1 and 4 days, respectively.

**Fig. 4:**
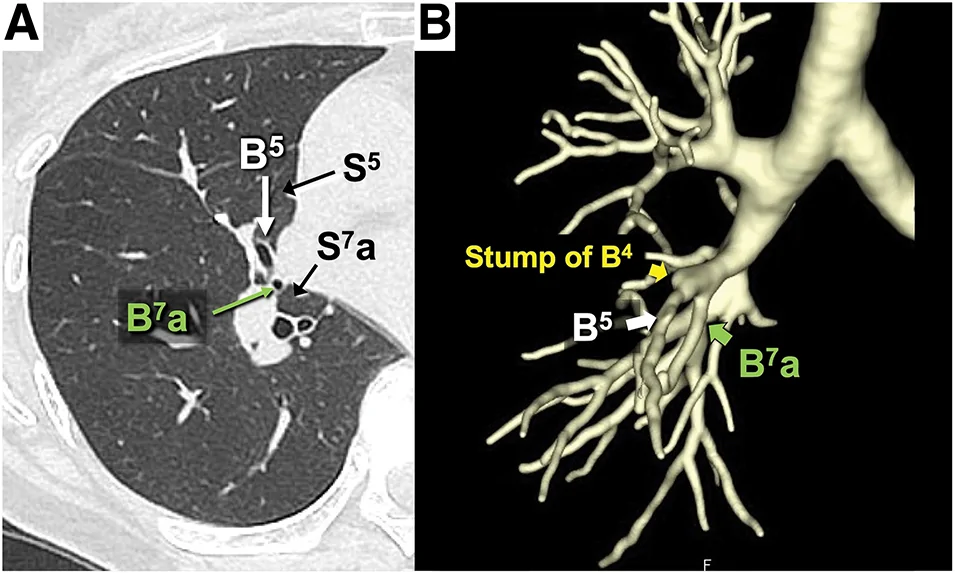
Postoperative CT and 3D reconstruction images. (**A**) S^5^ and S^7^a were preserved with the intact bronchi. (**B**) B^5^ and B^7^a were intactly preserved.

Postoperative percutaneous oxygen saturation increased from 94% to 98%, and the patient experienced improved exertional dyspnea. Adjacent residual segments (S^5^ and S^7^a) with intact segmental bronchi were preserved, as confirmed on the postoperative CT (**Fig. 4A** and **4B**).

## DISCUSSION

Although PAVFs are often treated by embolizations using some coils, which is minimally invasive, wedge resection, segmentectomy, or lobectomy can also be selected as a treatment approach. In this case, the embolization required many coils owing to the dilated vessels; the PAVF had a possibility of recurrence, and long-term follow-up would have been necessary.^9, 10)^ In contrast, segmentectomy totally removed the PAVF involving the dilated vessels, eliminating the risk of recurrence and the need for long-term follow-up.^11)^ Although wedge resection was first considered as a simple procedure among the surgical approaches, segmentectomy was finally selected because the PAVF, feeding artery, and drainage vein were totally contained within S^4^. If wedge resection was selected, there were concerns that the dilated feeding artery and vein would remain in the target segment, and long-term follow-up of the patient would have been required because of the risk of recurrence. A standard procedure for PAVF has not been established, and the appropriate treatment must be selected in each individual patient. While various endovascular interventions have been actively performed and developed as the minimally invasive treatment, surgical approaches should be reconsidered as a traditional curative treatment option, reducing the long-term follow-up for PAVF cases. On performing the surgery, we technically followed a specific procedural sequence to avoid cerebral infarction from emboli: V^4^ and A^4^ were divided sequentially after complete vascular dissection.

To the best of our knowledge, reports describing thoracoscopic right S^4^ segmentectomy for PAVF are extremely limited.^2)^ Although middle lobe segmentectomy has been selectively performed in malignant cases owing to technical improvements in thoracoscopic segmentectomy,^12)^ we believe that it can be appropriately considered for benign diseases.

Currently, we attempt to routinely perform a preoperative bronchial CT simulation and identify the target bronchus using ICG in all cases with a planned segmentectomy after our previous report.^8)^ Identifying the target bronchus during thoracoscopic segmentectomy can be challenging. Although intraoperative bronchial confirmation is traditionally performed using bronchoscopy, it requires technical expertise and is difficult with thinner bronchi owing to occlusion by sputum. Therefore, recognizing the necessity of a more reliable method, we devised an NIF imaging method for identifying the target segmental bronchus in thoracoscopic segmentectomy for tumors suspicious for malignancy. We successfully performed our NIF imaging method in various types of segmentectomy.^13)^ Other methods using NIF images for the identification of the target segmental bronchi have been reported; each method has advantages and disadvantages. Although our technique has the advantage of marking only the selected area of the target bronchus with a little amount of ICG and without spreading to the adjacent segmental bronchi, it may be technically difficult compared with other published methods that spray the ICG onto the target bronchus.^14–16)^ Based on the successful application of our technique in malignant diseases, NIF imaging was first applied in the present case of a benign disease.

However, transbronchial ICG administration has not been approved by the Japanese insurance system. Therefore, prior to the application of this method, practical trials were performed using a resected lung specimen to confirm the imaging of the target segmental bronchus. This method was commenced after obtaining approval from the Institutional Review Board. In this case, the preoperative bronchial CT simulation facilitated the recognition of an aberrant bronchus (B^7^a) diverging from the middle lobe bronchus, and the target segmental bronchus could be reliably identified under NIF imaging without intraoperative confusion. Without such preoperative simulation, the B^7^a might not have been identified as the bronchial branching pattern was extremely rare and not among the middle lobe branching patterns reported in recent 541 cases.^17)^

Thoracoscopic S^4^ segmentectomy was successfully performed for the first time, to the best of our knowledge, using NIF imaging with ICG to identify the target segmental bronchus while preserving the rare aberrant bronchus via preoperative bronchial simulation.

## CONCLUSIONS

Segmentectomy can play a curative role as complete resection for PAVF; the preoperative bronchial CT simulation was helpful for recognizing the variant bronchus, and the NIF imaging can help identify not only the intersegmental plane but also the target bronchus for division in PAVF case with an aberrant bronchus requiring thoracoscopic segmentectomy.

## SUPPLEMENTARY MATERIAL

Video 1 Bronchoscopic marking of B^4^ with ICG-soaked cotton and thoracoscopic right S^4^ segmentectomy involving an aberrant bronchus for PAVF using NIF images.
ICG, indocyanine green; NIF, near-infrared fluorescent; PAVF, pulmonary arteriovenous fistula

## DECLARATIONS

### Availability of data and materials

The data underlying this article will be shared on reasonable request to the corresponding author.

## References

[1] Lu H, Wang QG. Pulmonary arteriovenous fistula: diagnosis, interventional therapy and clinical progress. J Cardiothorac Surg 2026; 21:339. 10.1186/s13019-026-04224-4 PMC13248248 42035182

[2] Biçakçioğlu P, Gülhan SŞ., Sayilir E, et al. Surgical treatment of pulmonary arteriovenous malformations. Turk J Med Sci 2017; 47:161–6. 10.3906/sag-1509-30 28263484

[3] Falk AR, Nitsche LJ, Bontrager CE, et al. Surgical resection of diffuse pulmonary arteriovenous malformations (PAVMs). JTCVS Open 2024; 23: 309–17. 10.1016/j.xjon.2024.11.002 PMC11883706 40061558

[4] Schröder C, Fröhlich G, Harms CP, et al. Fistulectomy as an alternative to segmentectomy for pulmonary arteriovenous fistula. J Thorac Cardiovasc Surg 2002; 122:386–8. 10.1067/mtc.2001.113742 11479517

[5] Saji H, Okada M, Tsuboi M, et al. Segmentectomy versus lobectomy in small-sized peripheral non-small-cell lung cancer (JCOG0802/WJOG4607L): a multicentre, open-label, phase 3, randomised, controlled, non-inferiority trial. Lancet 2022; 399:1607–17. 10.1016/S0140-6736(21)02333-3 35461558

[6] Altorki N, Wang X, Kozono D, et al. Lobar or sublobar resection for peripheral stage IA non-small-cell lung cancer. N Engl J Med 2023; 388:489–98. 10.1056/NEJMoa2212083 PMC10036605 36780674

[7] Brunelli A, Decaluwe H, Gonzalez M, et al. European Society of Thoracic Surgeons expert consensus recommendations on technical standards of segmentectomy for primary lung cancer. Eur J Cardiothorac Surg 2023; 63:ezad224. 10.1093/ejcts/ezad224 37267148

[8] Kato H, Abiko M, Sato K. New technique for endobronchial targeting of the segmental bronchus during thoracoscopic segmentectomy. Interdiscip Cardiovasc Thorac Surg 2025; 40:ivaf038. 10.1093/icvts/ivaf038 PMC11997761 40036784

[9] Remy-Jardin M, Dumont P, Brillet PY, et al. Pulmonary arteriovenous malformations treated with embolotherapy: helical CT evaluation of long-term effectiveness after 2-21-year follow-up^1^. Radiology 2006; 239:576–85. 10.1148/radiol.2391050333 16484354

[10] Hering K, Sutphin P, Kalva S. Posttreatment monitoring of pulmonary arteriovenous malformations: challenges and approaches. Chest 2025; 168:1471–80. 10.1016/j.chest.2025.06.014 40543746

[11] Reichert M, Kerber S, Alkoudmani I, et al. Management of a solitary pulmonary arteriovenous malformation by video-assisted thoracoscopic surgery and anatomic lingula resection: video and review. Surg Endosc 2016; 30:1667–9. 10.1007/s00464-015-4337-0 26156615

[12] Zhang M, Wu QC, Ge MJ. The VVBA method for thoracoscopic right middle lobe segmentectomy. Gen Thorac Cardiovasc Surg 2021; 69:175–7. 10.1007/s11748-020-01398-z 32488833

[13] Kato H, Abiko M, Sato K. A near-infrared fluorescent imaging method for identifying the target segmental bronchus in thoracoscopic anatomical segmentectomy. JTCVS Tech 2025; 31:179–81. 10.1016/j.xjtc.2025.04.001 PMC12237857 40641765

[14] Gómez-Hernández MT, Forcada C, Rivas C, et al. Techniques for intraoperative identification and confirmation of segmental bronchus in minimally invasive segmentectomy. Video-assist Thorac Surg 2024; 9:25.

[15] Xu H, Wu X, Zhao S, et al. Indocyanine green nebulization visualizes the pulmonary bronchus during video-assisted thoracoscopic surgery. J Cardiothorac Surg 2025; 20:113. 10.1186/s13019-024-03130-x PMC11786342 39893458

[16] Takamori S, Nakatsuka M, Watanabe H, et al. Segmental bronchial marking method using indocyanine green for safe thoracoscopic segmentectomy: a single-center early experience. J Thorac Dis 2025; 17:2958–66. 10.21037/jtd-2025-242 PMC12170054 40529742

[17] Zhao B, Wu W, Duan G. Observation of bronchial anatomy and variation of the middle lobe of the right lung based on three-dimensional reconstruction of lung CT. J Cardiothorac Surg 2025; 20:172. 10.1186/s13019-025-03402-0 PMC11963428 40176074

